# Enhanced Polar Auxin Transport and Reduced Brassinosteroid Activity Drive Internode Elongation in Chinese Fir (*Cunninghamia lanceolata*)

**DOI:** 10.3390/plants15091411

**Published:** 2026-05-05

**Authors:** Chao Wu, Fang-Fang Wang, Fang-Fang Ma, Ling-Peng Ye, Shi-Yan Mu, Ya-Ting Yang, Xiao-Yu Qu, Ya-Ling Zhang, Shu-Bin Li, Shan-Shan Xu, Xiang-Qing Ma, Guang-Qiu Cao, Si-Zu Lin, Yu Chen

**Affiliations:** 1 Key Laboratory for Forest Adversity Physiological Ecology and Molecular Biology, Fuzhou 350002, China; 2 College of Computer and Information Science, Fujian Agriculture and Forestry University, Fuzhou 350002, China; 3 College of Forestry, Fujian Agriculture and Forestry University, Fuzhou 350002, China

**Keywords:** apical dominance, Chinese fir, endogenous hormones dynamics, gene regulation, internodal length, lateral bud initiation

## Abstract

Knot-free timber production in *Cunninghamia lanceolata* depends critically on internodal characteristics, yet the mechanisms governing internode elongation remain poorly understood, hindering breeding efforts for longer-internode varieties. In this study, we selected two clones with distinct internodal traits (the C1 clone exhibited a 25.03% longer internodal length than the C11 clone) as materials. Enzyme-linked immunosorbent assay (ELISA) and RNA sequencing were used to investigate dynamics in endogenous hormones and transcriptional regulation in internodal growth. Results showed that the difference in indole-3-acetic acid (IAA) rhythms in apical buds is a key factor of C1’s longer internodal growth; higher levels of IAA and cytokinins in the apical buds of C1 may support sustained internodal growth; upregulated IAA-related genes in upper phloem (*PIN1* and *SAURs*), which are involved in polar transport and signal response, indicates a stronger capacity to establish apical dominance. Hormone transport may be regulated by very long-chain fatty acids (VLCFAs). Consistent with reduced brassinosteroid activity, genes involved in VLCFA biosynthesis and transport were generally lower in C1, implying excessive VLCFA accumulation in C11 may be negative to IAA transporting and internode growth. This study offers a preliminary insight into internodal growth mechanisms influenced by hormone biosynthesis and transport in *C. lanceolata*., providing a basis for genetic improvement, germplasm selection, and exogenous hormone applications in knot-free timber cultivation.

## 1. Introduction

Chinese fir (*Cunninghamia lanceolata*), a well-known timber wood species with a cultivation history spanning more than a thousand years, is predominantly found in southern China and northern Vietnam [1]. Known for its rapid growth, high yield, and decay resistance, *C. lanceolata* covers an area of 8.95 million hectares in China, contributing to 625 million cubic meters in standing volume. It accounts for 25% of the country’s total commercial timber supply annually [1,2,3,4] and serves as the primary raw material in key industries such as construction, furniture manufacturing, and shipbuilding. However, as market demands evolve, the evaluation of wood value is no longer based solely on yield; quality factors such as the aesthetics of wood grain and workability are also taken into account, particularly in high-end markets where these attributes are highly valued. This shift in preferences highlights certain drawbacks of *C. lanceolata*, especially its prevalent knots, which not only affect its appearance but also impair its workability. The prevalence of knots in *C. lanceolata* wood is primarily due to the persistent nature of its lateral branches, which resist natural shedding [1]. Dead branches often remain on the tree for 5 to 8 years (Figure S1), becoming enveloped by the trunk as it grows [5], thereby generating numerous knots. To meet market preferences for knot-free wood, strategic measures such as the proactive removal of persistent branches are effective but require considerable labor and financial resources. Therefore, developing a practical and cost-effective approach to minimize knot formation in *C. lanceolata* wood is essential.

Selecting breeding materials with fewer lateral branches or longer internodes is a promising strategy to improve branching architecture for knot-free timber production. Internode length is influenced by both apical growth and lateral bud outgrowth [6,7]. The overall crown architecture of a tree is primarily shaped by the degree of apical dominance, which prompts apical growth and inhibits lateral bud activity. Axillary meristems are clusters of cells capable of forming new branches, located at the leaf axils (the junction between the leaf blade and the stem) [8,9]. These meristem cells can develop into fully formed branches under suitable environmental conditions, such as light, temperature, and soil nutrients [10,11,12]. When plants perceive these environmental cues, they trigger physiological responses that promote the growth of lateral buds into branches [13]. Hormonal interactions and their associated genetic signaling pathways are crucial in regulating internodal growth and lateral bud initiation [14]. Among these, auxin serves as a key regulator [12,15], specifically through its role in establishing a concentration gradient that enforces apical dominance and suppresses the activation of lateral buds [16]. Research indicates that reducing indole-3-acetic acid (IAA) synthesis and disrupting apical dominance can lead to increased lateral branch growth. Auxin’s influence extends to regulating lateral development by either suppressing cytokinin (CTK) synthesis [17,18] or enhancing strigolactone (SL) production [19,20], which act as downstream signaling molecules. CTKs contribute to lateral bud growth by boosting the expression of genes like *WUS*, which in turn enhances invertase activity and cell cycle gene expression, increasing the bud’s ability to attract resources [21,22]. SLs, predominantly produced in roots, mediate signals between the plant’s above-ground and below-ground parts [23]. When SLs reach the aerial tissues, they inhibit lateral bud growth by promoting the expression of *BRC1* [24], through mechanisms involving the F-box protein MAX2 [25,26], SPL transcription factors [27,28], and proteins such as D53, which negatively regulates SL signaling and can be modulated by CTKs [29]. Brassinosteroids (BRs) also play a crucial role in regulating lateral bud sprouting by modulating the effects of apical dominance and interacting with CTKs and abscisic acid (ABA) to influence auxin pathways [30,31]. They affect key genes involved in bud sprouting, such as *BZR1* and *BES1* [32], or influence cell wall loosening and microtubule organization to affect cell differentiation, division, and elongation, thereby facilitating lateral bud growth [30]. Additionally, gibberellins (GAs) and ABA are believed to influence lateral bud development [33,34].

Although understanding these hormonal interactions is essential for comprehending how plant architecture is influenced, research on hormonal dynamics and transcriptional regulation during internodal growth in *C. lanceolata* remains largely unexplored. The genes and pathways underlying internode length and lateral bud outgrowth, hidden behind the façade of endogenous hormone regulation, remain unknown. This gap poses a challenge for advancing wood quality improvements through the regulation of lateral branch growth. In this study, we examined two clones that exhibit distinctly different internode growth characteristics and performed a comparative analysis of dynamic levels of four key hormones (IAA, CTKs, SLs, and BRs) and transcriptional profiles in the apical bud (AB), lateral bud (LB), and upper phloem (UP) near the apical bud during internode elongation. We aim to shed light on the dynamics of hormonal interactions and associated transcriptional regulation in apical and lateral buds and their roles in shaping internode growth patterns, an area that has not been systematically studied in *C. lanceolata*. This preliminary research offers a reference point for future studies on lateral branch regulation and could support the selection of optimal germplasm resources. Additionally, it may inform the application of exogenous hormone treatments [35] to delay lateral bud initiation, promote internode elongation, and ultimately facilitate the production of high-quality, knot-free timber of *C. lanceolata.*

## 2. Materials and Methods

### 2.1. Experimental Site Overview

This study was conducted in a *C. lanceolata* superior clonal test forest at the Shandou section of Youxi State-owned Forest Farm in Fujian Province, China. The experimental forest is located in Tangchuan Township, Youxi County, Sanming City, Fujian Province, at approximately 118.510° E longitude and 26.165° N latitude (Figure S2), with an elevation of 1000 m. Situated in the hilly and mountainous region of central-western Fujian, the area experiences a subtropical monsoon climate, characterized by warm temperatures and abundant rainfall. The soil is acidic mountain red soil, with a pH between 4.0 and 5.0. The clonal test forest was established in April 2018, consisting of three replicated sites. Each site contains 13 *C. lanceolata* superior clones.

### 2.2. Selection of Sampling Individuals

In each site, 10 individuals per clone were randomly selected, resulting in a total of 30 individuals surveyed per clone, and 390 individuals in total across all 13 clones (Table S1). Based on a thorough analysis of growth and branching characteristics, two clones (C1: Yang061 and C11: YueTL5) with notable differences were chosen for further study (Figure S3).

### 2.3. Measurement of Growth and Branching Traits

The assessment of growth traits was carried out between February and March 2022, prior to the sprouting of apical buds in *C. lanceolata*. Growth measurements included tree height, diameter at breast height (DBH), and crown width for each individual. Branching traits recorded included the number of branch whorls, the number of lateral branches, average internodal length, average lateral branch length, basal diameter of lateral branches, and average branch angle. Additionally, the number of lateral branches per meter of the main trunk was calculated, along with the total basal cross-sectional area of lateral branches per meter of trunk, which was determined by summing the circular areas (using the basal diameter) of all lateral branches on each individual, reflecting the potential number and size of knots that could form in future logs.

### 2.4. Endogenous Hormone Level Analysis

To further validate the regulatory roles of IAA on internode elongation. We employed two clones with significant differences in branching traits, C1 and C11 (Figure S3), to join the measurement of the dynamic changes in endogenous hormone levels. Sampling began after the initiation of apical bud sprouting and was conducted every 7 days, with the collection time set between 8:00 and 10:00 a.m. on the day of sampling. For each sampling, three individuals were randomly chosen from each clone. Collected materials included apical buds (AB), upper phloem (UP) adjacent to the apical buds (if the main stem exceeded 3 cm for adequate sample quality), and lateral buds (LB). Given the variations in growth rates and lateral bud emergence among individuals, the specific tissues were collected according to their development at the time of sampling. The final collection occurred when lateral bud emergence was observed in the last selected individual.

Levels of endogenous hormones, including IAA, CTKs, SLs, and BRs, were quantified using enzyme-linked immunosorbent assay (ELISA) kits (Yuanju Biotechnology Center, Shanghai, China). The measurements were carried out as follows: the collected tissue samples of *C. lanceolata* were ground into fine powder using liquid nitrogen in a pre-chilled mortar. An aliquot of 0.1 g of the powder was weighed, mixed with pre-chilled phosphate-buffered saline (PBS), and extracted at 4 °C for 3 h. After centrifugation, the supernatant was collected as the test sample. On a microplate, blank wells, standard wells, and sample wells were set up. No sample or enzyme-labeled reagent was added to the blank wells. Standard solutions were added to the standard wells. For the sample wells, the sample diluent was added first, followed by the test sample, and the mixture was homogenized. The plate was sealed and incubated at 37 °C for 30 min, followed by five washes. Enzyme-labeled reagent was then added to all wells except the blank wells, and the plate was incubated again at 37 °C for 30 min, followed by another five washes. Chromogenic solutions A and B were added, and the plate was incubated in the dark for 10 min. The reaction was stopped by adding stop solution. The absorbance was measured at 450 nm using a microplate reader, with the blank well used as a zero reference. A standard curve was constructed based on the absorbance of the standard solutions. The absorbance of the sample wells was interpolated into the standard curve to calculate the concentration, which was then multiplied by the dilution factor to obtain the actual hormone content in the sample.

### 2.5. Exogenous Plant Hormone Experiment

The exogenous plant hormone experiment was conducted from 1st July to 28th November 2025. Forty-eight one-year-old clonal cuttings for each clone, C1 and C11, with good growth status, were selected for each treatment. Six treatments were set for each clone, including low concentration IAA (LIT, Low IAA Treat, 50 mg·L^−1^), high concentration IAA (High IAA Treat, HIT, 150 mg·L^−1^), low concentration IAA inhibitor (Low TIBA Treat, LTT, 50 mg·L^−1^), high concentration IAA inhibitor (High TIBA Treat, HTT, 150 mL·L^−1^), water treat (WT), and solvent treat (ST). Each treatment consisted of four biological replicates (individual plants). The treatments were applied to the apical buds every seven days at 9:00 a.m., with 5 mL per plant per application. Due to the small size of the apical bud (AB) tissue, the samples were divided into the pre-treatment group and the post-treatment group. For the pre-treatment group, apical buds (AB) and the upper phloem (UP) adjacent to the apical buds (AB) were collected before the start of the experiment (1 July), and their internode distance was measured. On 1 December, the apical buds (AB) and upper phloem (UP) of the post-treatment group were collected, and the internode distance was measured. The contents of IAA and IAA oxidase in the samples were determined using the same method as in 2.4. The IAA oxidase was measured using the Indole Acetic Acid Oxidase activity monitoring kit (Solarbio, Beijing, China). The measurements were carried out according to the manufacturer’s protocol.

### 2.6. RNA Extraction

Due to the variation in lateral bud emergence timing between the two clones, samples for transcriptome analysis were collected at the onset of lateral bud appearance (C1: 21 days after initiation of apical bud sprouting, C11: 28 days after initiation of apical bud sprouting), simultaneously from the individuals that were used for measuring endogenous hormones. The collected tissues included ABs, UPs, and LBs, which were mentioned previously. Three biological replicates of each tissue type were gathered from each clone, resulting in 18 samples in total. Immediately after collection, samples were frozen in liquid nitrogen and transported to the laboratory, where they were stored at −80 °C until total RNA extraction.

Total RNA was extracted from the samples using the RNAprep Pure Plant Kit (Tiangen, Beijing, China). The concentration, purity, and integrity of the RNA were evaluated using a NanoDrop 2000 spectrophotometer (Thermo Fisher Scientific, Wilmington, DE, USA) and the RNA Nano 6000 Assay Kit (Agilent Technologies, Santa Clara, CA, USA), respectively.

### 2.7. RNA Sequencing

Once the samples passed quality control, sequencing libraries were prepared using the Hieff NGS Ultima Dual-mode mRNA Library Prep Kit for Illumina (Yeasen Biotechnology, Shanghai, China). The libraries were then sequenced on the Illumina NovaSeq platform, generating 150 bp paired-end reads. The sequencing was conducted by Biomarker Technologies Corporation (Beijing, China).

### 2.8. Annotation and Differentially Expressed Genes Screening

The clean data were aligned to the *C. lanceolata* reference genome for further analysis and annotation, with the reference genome information sourced from Lin et al.’s study [3]. The Hisat2 software v2.0.4 (https://github.com/DaehwanKimLab/hisat2, accessed on 13 June 2024) was used to perform the alignment with the reference genome [36]. Gene function annotation was conducted by sequence alignment using the following databases: Nr (NCBI non-redundant protein sequences, ftp://ftp.ncbi.nih.gov/blast/db, accessed on 15 June 2024) [37], Pfam (Protein family, https://pfam.xfam.org/, accessed on 15 June 2024) [38], Swiss-Prot (A manually annotated and reviewed protein sequence database, https://www.uniprot.org/, accessed on 15 June 2024) [39], KO (KEGG Ortholog database, https://www.kegg.jp/, accessed on 15 June 2024) [40], and GO (Gene Ontology, https://geneontology.org/, accessed on 15 June 2024) [41]. Differential expression analysis was performed using the DESeq2 software v1.30.1 (https://bioconductor.org/packages/release/bioc/html/DESeq2.html, accessed on 15 June 2024) [42]. Gene expression levels were quantified using FPKM (Fragments Per Kilobase of transcript per Million mapped reads) values. Genes with a fold change ≥ 2 and a false discovery rate (FDR) < 0.05 were considered differentially expressed genes (DEGs). The KOBAS database (http://kobas.cbi.pku.edu.cn/, accessed on 15 June 2024) [43] and clusterProfiler software v4.4.4 (https://guangchuangyu.github.io/software/clusterProfiler/, accessed on 15 June 2024) [44] were employed to analyze the enrichment of DEGs in KEGG pathways. Information on all the DEGs is shown in Table S2.

### 2.9. WGCNA Network Construction

The WGCNA was conducted using the WGCNA package v1.72-5 (https://cran.r-project.org/web/packages/WGCNA/index.html, accessed on 30 June 2024) in R-Studio [45]. Genes with the top 75% median absolute deviation (MAD) of FPKM values were selected for network construction. The network was then visualized using Cytoscape v3.10.1 software (https://github.com/cytoscape/cytoscape/releases/3.10.1/, accessed on 30 June 2024).

### 2.10. Statistical Analysis

All statistical analyses were performed using R software (version 4.3.1) in R-Studio. An independent samples Student’s *t*-test was applied to compare the differences in growth parameters and endogenous hormone contents between the two clones with the *t*.test function.

Principal component analysis (PCA) was performed to visualize global gene expression variation between the two clones and verify biological replicate consistency. FPKM-normalized gene expression data were log_2_(x + 1)-transformed to reduce extreme value interference before PCA. The transformed data were mean-centered and scaled to unit variance per gene across all samples (center = TRUE, scale = TRUE), ensuring equal gene contribution and avoiding high-expression gene dominance. PCA was conducted via R’s stats::prcomp; PC1 and PC2 (highest variance explanation) were visualized with ggplot2 (v3.4.4), with variance percentages annotated on plot axes.

## 3. Results

### 3.1. Differences in Branching Traits

**Figure 1:**
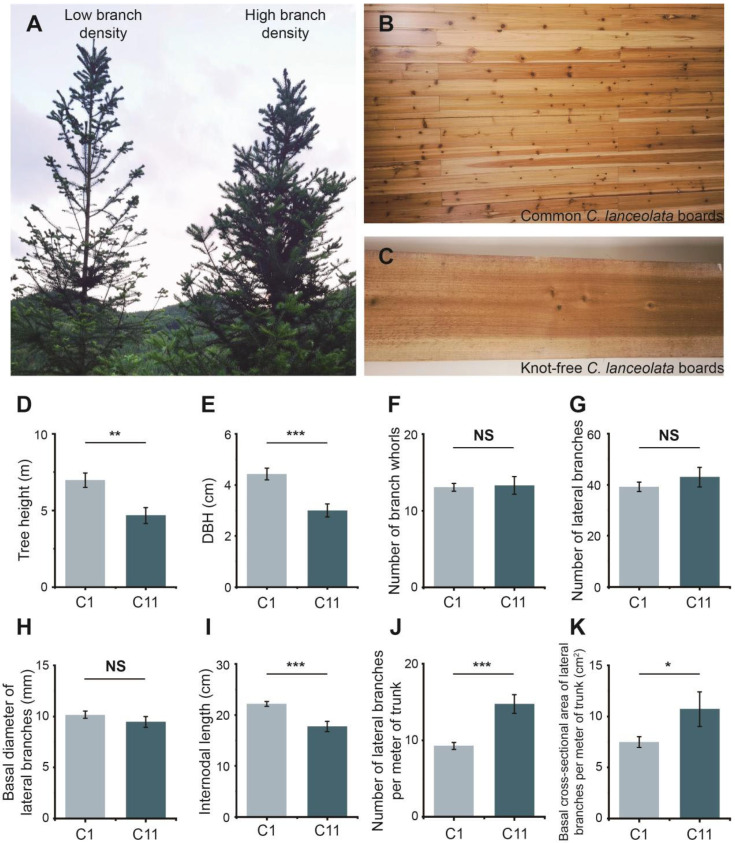
Comparative analysis of growth and branching traits between selected clones. An example of different branching traits in *C. lanceolata* (**A**). Comparison between common *C. lanceolata* boards (**B**) and knot-free board (**C**) highlights the impact of knot quantity on wood quality. Bar charts comparing the tree height (**D**), diameter at breast height (**E**), number of branch whorls (**F**), number of lateral branches (**G**), basal diameter of lateral branches (**H**), internodal length (**I**), number of lateral branches per meter of trunk (**J**), and basal cross-sectional area of lateral branches per meter of trunk (**K**) of the two selected clones is used to emphasize their performance differences. Significance levels were determined using an independent-sample t-test, with marked “NS”, “*”, “**” and “***” indicating *p* > 0.05, *p* < 0.05, *p* < 0.01, and *p* < 0.001, respectively. Bars indicate means  ±  SD.

The quantity of lateral branches (Figure 1A) directly impacts the number of knots in *C. lanceolata* timber, a key factor in evaluating timber quality (Figure 1B,C). In our comparison of growth and internodal traits across 13 clones, clone C1 stood out with superior performance, making it more suitable for producing timber with fewer knots. Conversely, clone C11 exhibited significantly different characteristics (Figure S2).

For growth traits, C1 had an average height (Figure 1D) and DBH (Figure 1E) that were 49.25% and 47.33% higher than C11, respectively, with both differences being statistically significant (*p* < 0.01). In terms of branching traits, there were no significant differences between the two clones in the number of branch whorls (Figure 1F), number of lateral branches (Figure 1G), or basal diameter of lateral branches (Figure 1H). However, C1 exhibited a 25.03% longer internodal length (Figure 1I), while the number of lateral branches per meter of trunk (Figure 1J) and the total basal cross-sectional area of lateral branches per meter of trunk (Figure 1K) were reduced by 37.14% and 69.87%, respectively. This indicates that timber from C1 may have significantly fewer knots and a smaller knot area upon harvest. In conclusion, these results demonstrate clear differences in internodal traits between clones C1 and C11, with C1 showing potential for higher wood quality.

### 3.2. Dynamic Changes in Endogenous Hormone Levels

**Figure 2:**
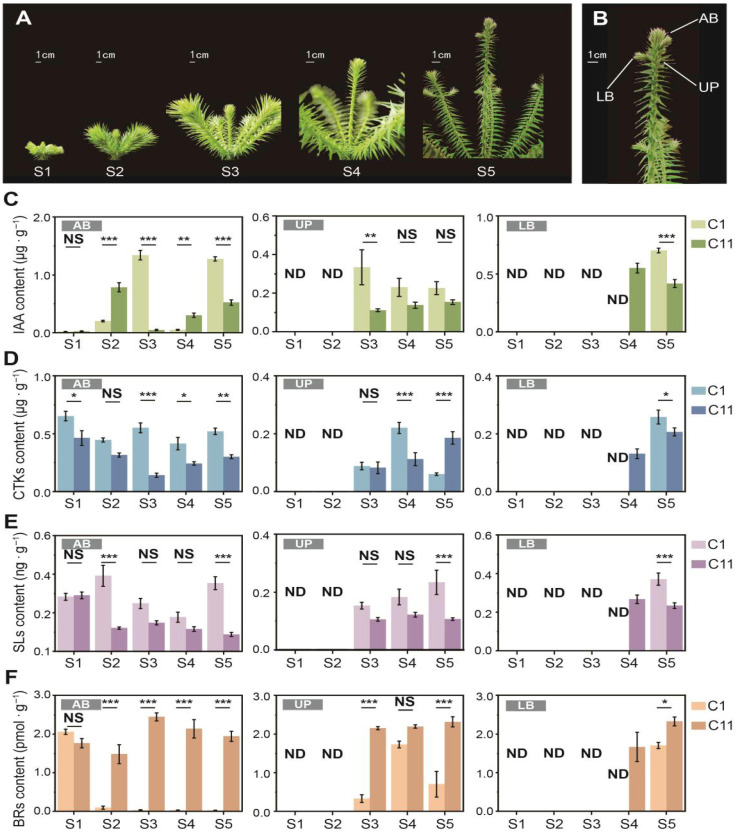
Sampling stages, tissue locations and the dynamic changes in endogenous hormone levels. Sampling was conducted from the onset of winter bud sprouting until the formation of the first internode (lateral bud appearance), covering 5 stages (**A**). Apical buds (AB), upper phloem (UP), and lateral buds (LB) were sampled for endogenous hormone measurements (**B**). However, not all tissues were present at every stage. The dynamic changes of IAA (**C**), CTKs (**D**), SLs (**E**), and BRs (**F**) in the different tissues are presented in bar charts and marked with “ND” if no available data were recorded. Each chart shows the fluctuations in hormone levels across the various tissues and stages. Significance levels were determined using an independent-sample *t*-test, with marked “NS”, “*”, “**” and “***” indicating *p* > 0.05, *p* < 0.05, *p* < 0.01, and *p* < 0.001, respectively. Bars indicate means  ±  SD.

Each spring, as the annual growth cycle of *C. lanceolata* begins, the lateral buds surrounding the apical bud are the first to break dormancy, initiating the development of lateral branches. These lateral buds are likely dormant winter buds formed in the previous year. After that, the apical bud also elongates gradually to form the main trunk. As the main trunk reaches a certain stage of growth, new lateral buds emerge in the cortex beneath the apical bud, leading to the formation of the first internode for that year’s growth (Figure 2A).

During this internodal growth process, significant differences in the dynamic changes in endogenous hormone levels were observed. The IAA, a hormone closely related to apical dominance, showed distinct patterns in both clones (Figure 2C). In the AB tissues, its levels were initially very low. Both clones followed a similar pattern of rising, falling, and then rising again, with significant differences (*p* < 0.01) at different stages. However, C1 exhibited a noticeable decrease at the S4 stage, later than clone C11. Simultaneously, higher levels of IAA in C1 at S3 (2805.12%) and S5 (144.03%) were recorded, respectively. Notably, a distinct drop in IAA levels in the AB tissues preceded the emergence of lateral buds in both clones. UP samples were collected from both clones at the S3 stage, while LB samples were gathered at the S4 stage for clone C11 and at the S5 stage for clone C1. Significant differences (*p* < 0.01) in IAA levels were observed in the UP tissues at S3 and in the LB tissues at S5 between the two clones.

At the S1 stage, both clones exhibited relatively high CTK levels in the AB tissues which then fluctuated and gradually declined (Figure 2D). Clone C1 consistently performed higher CTK levels than C11 during the S3 to S5 stages, with increases of 291.19%, 70.71%, and 73.13%, respectively. In the UP tissues, C1 showed 96.08% higher than C11 at the S4 stage but was surpassed by 208.67% at the S5 stage. A significant difference (*p* < 0.05) was also observed in the LB tissues at the S5 stage, where C1 was 25.02% higher.

At the S1 stage, no significant difference in SL content was observed in the AB tissues between the two clones. However, clone C1 followed a pattern of rising, falling, and then increasing again, while clone C11 showed a significant decrease at the S2 stage and remained relatively low afterward (Figure 2E). At the S2 and S5 stages, SL levels in the AB tissues of clone C1 were 225.62% and 303.66% higher than those in C11, respectively. From S3 to S5, SL content in the UP tissues of clone C1 gradually increased, while in clone C11 remained relatively low and stable. In the LB tissues at the S5 stage, SL content in clone C1 was 58.91% higher than that in C11.

In most tissues and sample stages, BR levels were lower in clone C1 (Figure 2F). At the S1 stage, no significant difference was observed in the AB tissues between the two clones. However, in the following stages, BR levels in C1 decreased to very low levels, while C11 maintained relatively higher levels. In the UP tissues, BR content in C1 initially increased before decreasing, resulting in no significant difference between the two clones at the S4 stage. In the LB tissues at the S5 stage, BR levels in C1 were significantly lower (*p* < 0.05) than those in C11.

### 3.3. Effects of Exogenous Hormone Treatments on Internode Elongation

**Figure 3:**
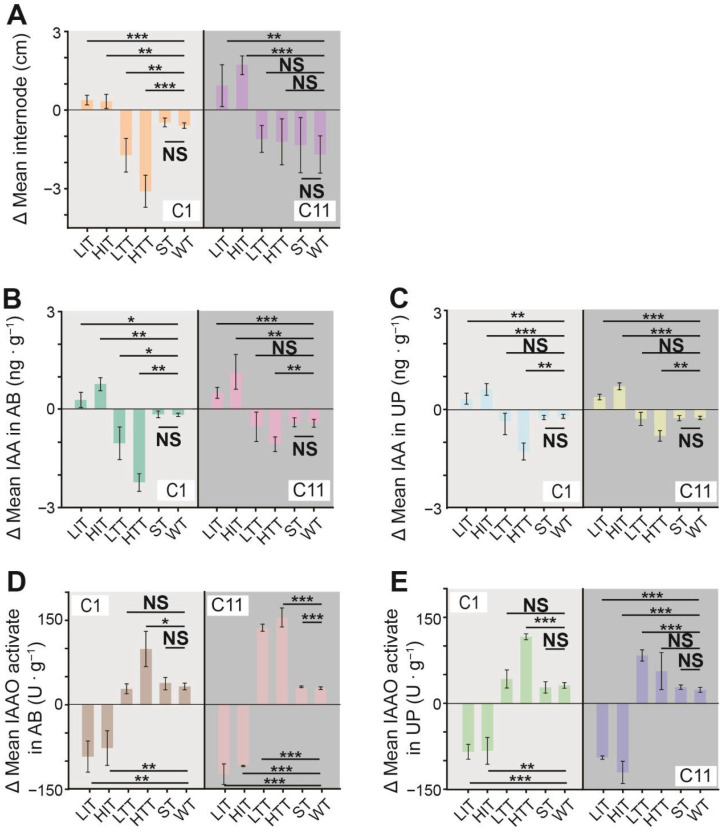
Effects of exogenous hormones on internode elongation growth. Clones C1 and C11 were treated with low and high concentrations of IAA(LIT/HIT) and TIBA(LTT/HTT), as well as with solvent (ST) and water (WT), respectively. Shown are the mean after–before differences (Δmean) for each clone, tissue, and treatment in: (**A**) internode length, (**B**) IAA content in AB tissues, (**C**) IAA content in UP tissues, (**D**) IAA oxidase activity in terminal AB tissues, and (**E**) IAA oxidase activity in UP tissues. Significance levels were determined using an independent-sample *t*-test, with marked “NS”, “*”, “**” and “***” indicating *p* > 0.05, *p* < 0.05, *p* < 0.01, and *p* < 0.001, respectively. Bars indicate Δmeans ± SD.

Compared with the control group (WT), exogenous IAA treatment significantly increased internode length in both clones, whereas TIBA treatment resulted in shortened internodes. However, the effect of exogenous IAA was stronger in clone C11 than in clone C1; similarly, exogenous TIBA appeared to have a more pronounced effect on clone C1 (Figure 3A). Following exogenous IAA treatment, IAA content in both AB tissues and UP tissues increased markedly in both clones, while TIBA treatment led to a decrease in IAA content. The effect of exogenous IAA was slightly stronger in clone C11 than in clone C1, whereas exogenous TIBA had a more pronounced effect on clone C1 (Figure 3B,C). After exogenous IAA treatment, IAA oxidase (IAAO) activity in AB tissues and UP tissues decreased markedly in both clones, whereas TIBA treatment resulted in increased IAAO activity. In AB tissues, the effects of both exogenous IAA and TIBA were stronger in clone C11 than in clone C1. In UP tissues, exogenous IAA had a slightly stronger effect on clone C11 than on clone C1; for exogenous TIBA, the effect was stronger in clone C11 under low-concentration treatment, but stronger in clone C1 under high-concentration treatment (Figure 3D,E).

### 3.4. Genes Expressed Patterns

**Figure 4:**
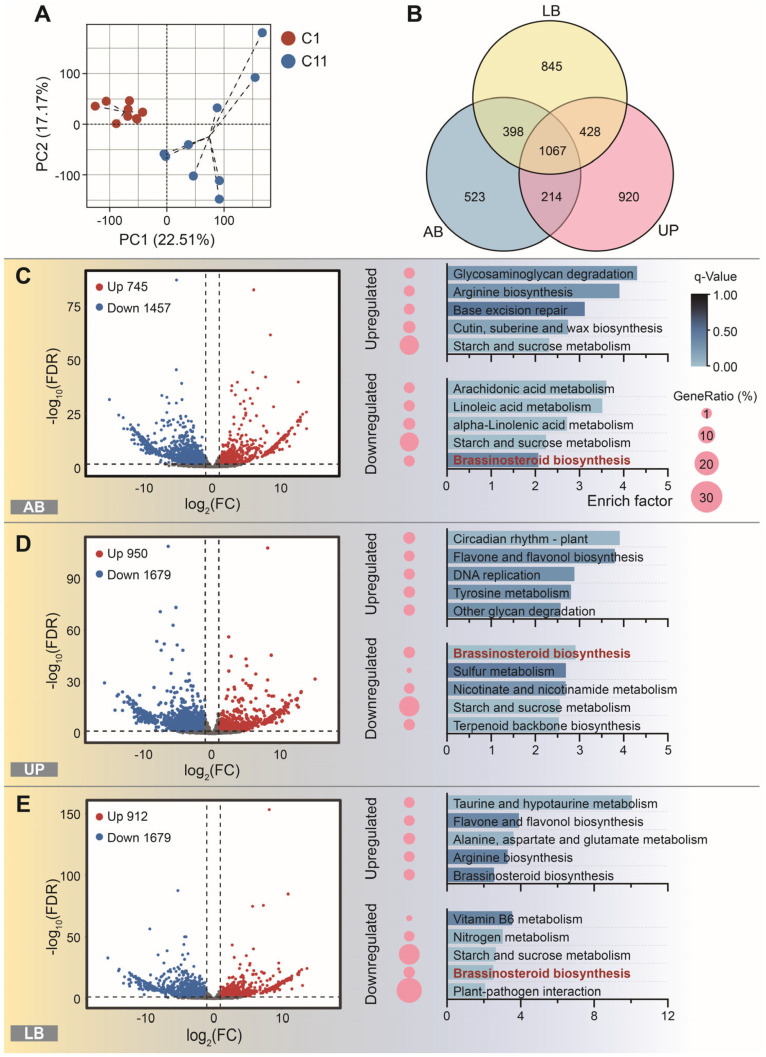
Analysis of RNA-seq results. The PCA highlights the gene expression differences between the two clones (**A**), and the Venn diagram shows the DEGs in the AB, UP, and LB tissues (**B**). Volcano charts present the number of upregulated and downregulated DEGs in AB (**C**), UP (**D**) and LB (**E**) tissue, along with KEGG enrichment analysis results, respectively. The bar chart displays the top 5 KEGG pathways with the highest enrichment levels for both upregulated and downregulated DEGs. The bar length represents the enrichment factor, while the color corresponds to the q-value. The size of the pink circles on the left of the bars indicates the GeneRatio for each respective pathway. The dotted lines in PCA plot (A) are used to highlight the grouping of the samples (C1, C11). The intersection points of the dotted lines represent the centers of each group of samples on a two-dimensional plane composed of PC1 and PC2. The vertical dotted lines in the volcano plot(C-E) represent the threshold of fold change of FPKM (|log_2_FC|>1), while the horizontal dotted lines represent the threshold of False Discovery Rate (FDR<0.05, -log_10_FDR>1.3)

Principal component analysis (PCA) based on gene expression data profiles revealed a clear separation of transcriptomic patterns between the two clones (Figure 4A). The first and second principal components accounted for 22.51% and 17.17% of the total variance, respectively. All biological replicates of C1 clustered tightly on the negative axis of PC1, whereas C11 replicates were distinctly distributed on the positive axis of PC1, with high reproducibility within each group. This suggests that distinct gene regulatory patterns between the two clones during lateral bud differentiation. Across all examined tissues, 1067 DEGs were shared by both clones. In addition, 523, 920, and 845 tissue-specific DEGs were identified in AB, UP, and LB, respectively (Figure 4B).

A total of 745, 950, and 912 upregulated genes, along with 1457, 1679, and 1826 downregulated genes, were identified in the AB, UP, and LB tissues, respectively (Figure 4C–E). KEGG enrichment analysis showed that the DEGs in the AB tissues were predominantly associated with the “glycosaminoglycan degradation”, “arginine biosynthesis”, “arachidonic acid metabolism”, “linoleic acid metabolism” and “base excision repair” pathway, while those in the UP tissues were mainly involved in the “circadiam rhythm-plant”, “flavone and flavonol biosynthesis”, “brassinosteroid biosynthesis”, “DNA replication” and “tyrosine metabolism” pathway, and the LB tissues were primarily linked to the “taurine and hypotaurine metabolism”, “flavone and flavonol biosynthesis”, “alanine, aspartate and glutamate metabolism”, “viamin B6 metabolism” and “arginine biosynthesis” pathway (Table S3).

**Figure 5:**
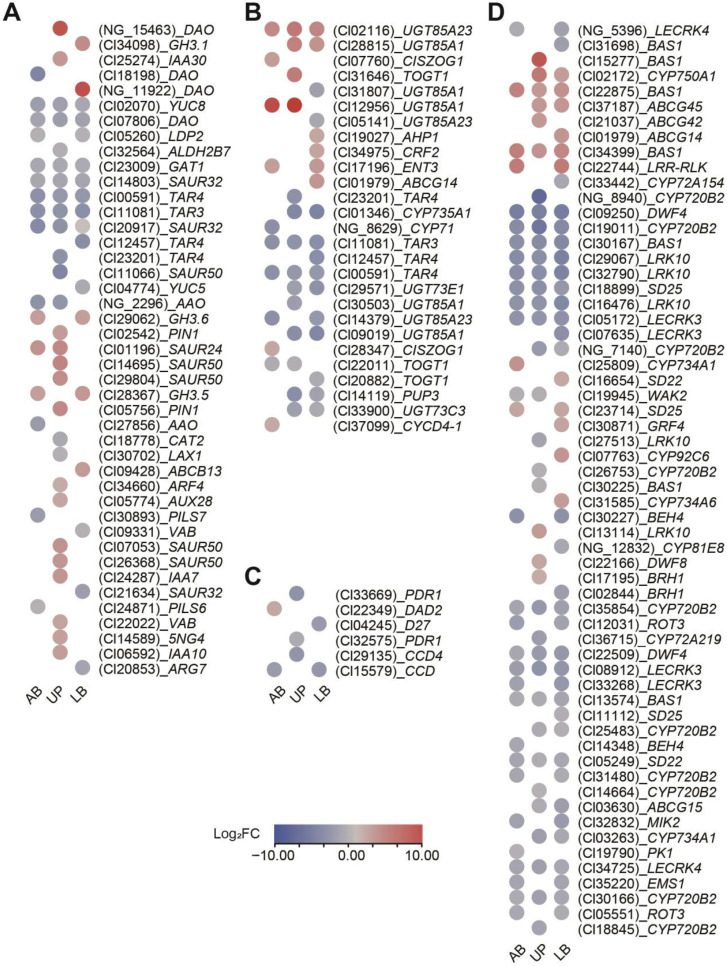
Expression levels of DEGs related to endogenous hormones. Expression pattern, including (**A**) auxin-, (**B**) cytokinin-, (**C**) strigolactone-, and (**D**) brassinosteroid-related DEGs, is exhibited. These genes are involved in biosynthesis, signal transduction, and degradation processes. The color and size of the circular cells represent the degree of upregulation or downregulation: red indicates upregulation, and blue indicates downregulation. The labels within the cells correspond to the gene IDs from the reference genome annotation (in parentheses) and their homologous gene names.

To further explore gene regulation related to the four previously detected endogenous hormones, we analyzed the expression levels of the DEGs associated with these hormones. A total of 43 auxin-related genes were identified (Figure 5A), with the *DAO* homolog gene significantly upregulated in both AB (NG_11922) and UP tissues (NG_15483). For cytokinin-related genes, 27 showed differential expression (Figure 5B), with a homolog of *UGT85A1* significantly upregulated in both UP and LB tissues (Cl12956). Relatively few DEGs related to strigolactones were identified, with only 6 detected, most of which were downregulated (Figure 5C). These included two *PDR1* homologs, two *CCD* homologs, and one *D27* homolog. The only upregulated gene was a *DAD2* homolog, observed in the LB tissues. Brassinosteroids had the longest list of related DEGs, with 60 identified, most of which were downregulated (Figure 5D). Notably, homologs of *CYP720B2* (Cl19011), *DWF4* (Cl09250), *BAS1* (Cl30167), *LRK10* (Cl29067, Cl16476 and Cl32790), and *SD25* (Cl18899) were consistently downregulated across all three tissues, while several homologs of *BAS1* (Cl15277, Cl22875 and Cl34399) showed higher levels of upregulation.

### 3.5. WGCNA Mining of Endogenous Hormone-Related Genes

**Figure 6:**
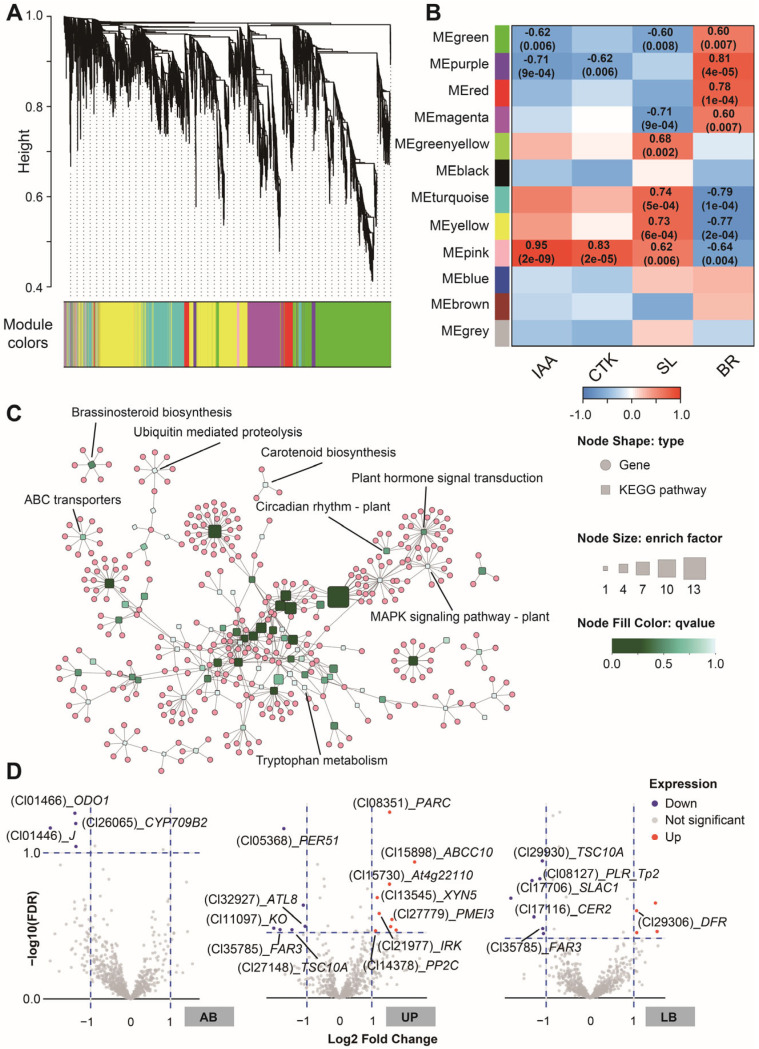
WGCNA based on the RNA-seq data and profiles in KEGG enrichment of the selected module. All expressed genes were clustered into 11 modules (**A**). Correlation analysis between these modules and the dynamic levels of IAA, CTKs, SLs, and BRs (**B**) revealed that the pink module had the strongest correlations. The KEGG enrichment analysis of the genes in the pink module is shown (**C**), where node shapes indicate types: circles represent genes, and rectangles represent KEGG pathways. The size of the rectangle nodes reflects the enrichment factor of the KEGG pathways, while the color represents the q-value. These parameters are used to assess the functional roles of genes within the pink module. Greater pathway enrichment suggests a higher concentration of regulatory genes influencing the associated biological processes. The volcano plot of gene expression in the pink module using FPKM values is represented (**D**). The vertical dotted lines in the volcano plot(D) represent the threshold of fold change of FPKM (|log_2_FC|>1), while the horizontal dotted lines represent the threshold of false discovery date (FDR<0.1, -log_10_FDR>1).

To investigate the relationship between endogenous hormones and gene expression during internodal growth, we conducted a Weighted Gene Co-expression Network Analysis (WGCNA) network. Hierarchical clustering divided all genes into 12 modules (including a gray module for unclustered genes, Figure 6A). Among these, the pink module showed the strongest correlations with the levels of the four endogenous hormones (Figure 6B), making it the primary focus of further analysis.

The pink module contained 981 genes, including 14 transcription factors (Table S4). KEGG enrichment analysis indicated that these genes were involved in hormone biosynthesis pathways such as “brassinosteroid biosynthesis”, “carotenoid biosynthesis”, and “tryptophan metabolism”, as well as hormone signaling pathways like “plant hormone signal transduction”, “circadian rhythm-plant”, “MAPK signaling pathway-plant”, and “ABC transporters”. However, the enrichment factors for these pathways were relatively low. More genes were enriched in pathways related to fatty acid metabolism, including “fatty acid elongation”, “biosynthesis of unsaturated fatty acids” and “fatty acid biosynthesis” (Figure 6C and Table S5). The differential expression patterns of the pink module genes in different tissues of the two clones are shown in Figure 6D. In AB tissues, three genes exhibited differential expression (Log2FC > 1, FDR < 0.1), all of which were downregulated. These included *ODO1* (Cl01466), which is involved in hormone signaling and metabolism, and *CYP709B2* (Cl26065), which participates in hormone metabolism and may alter IAA homeostasis. In the UP tissues, 15 genes showed differential expression, comprising nine upregulated and six downregulated genes in C1. Among the upregulated genes were *At4g22110* (Cl15730), involved in the metabolism of the auxin precursor tryptophan and stress response; *PP2C* (Cl14378), which affects IAA signal transduction; *PARC* (Cl08351), a cytokinin signaling-responsive gene; *PMEI3* (Cl27779), involved in cell wall remodeling and apical meristem development regulation; *IRK* (Cl21977), associated with somatic embryogenesis, brassinosteroid signaling, and maintenance of the apical meristem; *XYN5* (Cl13545), which promotes cell wall loosening and enhances IAA transport efficiency; and *ABCC10* (Cl15898), a member of the ABC transporter C family directly involved in transmembrane IAA transport. Among the downregulated genes were *FAR3* (Cl35785), involved in cutin and suberin synthesis, potentially affecting phloem barrier function and water/hormone transport, and *TSC10A* (Cl27148), which provides the precursor backbone for very long-chain fatty acid-containing sphingolipids. In LB tissues, 11 differentially expressed genes were identified, of which three were upregulated and eight downregulated in C1. The upregulated genes included *DFR* (Cl29306), which participates in flavonoid biosynthesis. The downregulated genes included *FAR3* (Cl35785), a lipid synthesis-related gene; *SLAC1* (Cl17116), an anion channel protein gene; *CER2* (Cl17116), a key enzyme involved in post-elongation modification of very long-chain fatty acids; *PLR_Tp2* (Cl08127), a key enzyme in the lignin biosynthesis pathway; and *TSC10A* (Cl29930).

**Figure 7:**
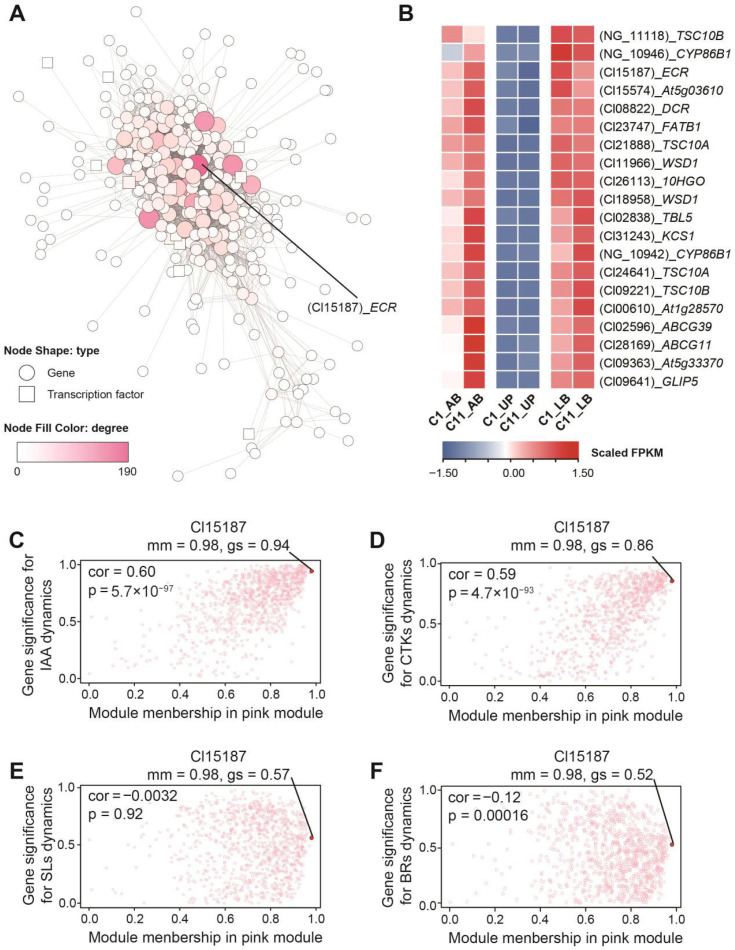
The pink module information of the gene co-expression network. The network structure of the pink module is represented (**A**). The node shapes indicate node types: circles represent genes, while rectangles represent transcription factors. Both the size and color of the nodes reflect the degree value of the corresponding gene, which is used to assess its connectivity within the network. Heatmap of the expression levels of the top 20 genes with the “degree” attribute in the pink module across the three tissues (**B**). Scatter plots depict gene significance for IAA (**C**), CTKs (**D**), SLs (**E**) and BRs (**F**) dynamic content vs. module membership in the pink module.

Upon mining the network structure of the pink module (Figure 7A), we found that most of the top 20 genes ranked by degree were homologs of genes involved in the synthesis, modification, and transport of very long-chain fatty acids (VLCFAs) (Figure 7B, Table S6). Among them, a homolog of *ECR* (Cl15187) has the highest connective degree (Table S6), indicating its frequent interactions with other genes and its potential role as a key regulatory hub in the module. Further analysis revealed that Cl15187 had a high gene module membership (mm) value of 0.98 and gene significance (gs) for trait correlation values above 0.5, indicating its expression pattern is closely aligned with other genes in the module and inversely correlated with the dynamic changes in the detected endogenous hormone levels, particularly in IAA and CTKs (Figure 7C–F). This suggests that Cl15187 may play a significant role in the hormone-regulated network influencing internodal growth via VLCFAs in *C. lanceolata*.

## 4. Discussion

### 4.1. Internode Growing Characteristics Fit for Knot-Free Timber Production of C. lanceolata

Branching is essential for most trees as it provides the structure for leaves, which serve as the “factories” of photosynthesis by capturing sunlight [11]. To maximize light absorption, trees employ two strategies: vertical growth of the main trunk to access higher light levels and horizontal expansion through lateral branches to increase surface area [46]. However, when human needs conflict with these natural growth strategies, we tend to favor traits that serve our purposes. In timber production, particularly for knot-free wood, vertical growth is prioritized over lateral branching [47]. This strategy is often emphasized in tree breeding programs aimed at producing high-quality, knot-free timber.

Our research aims to explore the regulation governing lateral bud growth, with the goal of understanding the lateral bud initiation and internodal growth of *C. lanceolata* initially, and certain patterns emerged. Clone C1, which exhibited a lower potential knot density, showed a delayed onset of the first round of lateral bud formation compared to clone C11, and contributed to longer internode (Figure 2C–F). While with short-term observation this may seem a minor difference, this could accumulate over time, leading to significant differences by the end of a full rotation period. Additionally, the significant difference in internodal length (Figure 1I) suggests that trunk growth rate plays a more crucial role in determining knot density. This indicates that the activity of the apical bud, which regulates the timing of lateral bud emergence, may be a key factor driving the internode growing process.

Our study only encompasses two phenotypic types, representing a small subset of the extensive variation in branching patterns found in *C. lanceolata*. Among these, the phenotype with longer internode length shows potential as a valuable breeding resource for producing knot-free timber. However, other germplasm resources with diverse branching traits also exist. For example, some clones naturally produce fewer branches. Yang et al. [48] have identified a unique variant known as the “DUGAN” fir, which has almost no lateral branching. Although its high susceptibility to lodging makes it unsuitable for timber production, it serves as an important resource for mechanistic studies. Nevertheless, the limited number of clones in this study constrains the ability to draw more definitive and broadly applicable conclusions. Future research should expand the scope by including a wider range of phenotypic variations for a more comprehensive analysis.

On the other hand, it is important to recognize that modern plantations are not solely focused on economic gains; ecological functions must also be taken into account. When selecting resources with fewer branches as primary cultivation targets, there may be concerns about a potential reduction in overall carbon sequestration efficiency [49]. This issue requires long-term observation and further evidence. Ultimately, selecting resources that balance both economic and ecological benefits is crucial for maximizing overall outcomes.

### 4.2. Differences in Auxin Rhythms May Play One of the Essential Roles of Variations in Internodal Growth

Endogenous hormone levels are crucial for lateral bud formation and lateral branch growth. Our results revealed distinct hormonal patterns across different tissues. IAA, a key signal regulating apical bud growth and lateral bud formation, may play a central role [50]. Through the polar transport of IAA, plants establish concentration gradients that inhibit lateral bud growth, creating apical dominance [12]. In both clones, we observed similar patterns of accumulation in AB tissues: IAA levels rose after apical buds sprouting and sharply declined just before lateral bud emergence (Figure 2C). This suggests that the apical bud actively releases apical dominance. However, in clone C1, this release was delayed, and the changes were more pronounced, potentially explaining its longer internodal growth before lateral bud formation.

At the gene expression level, the UP tissue exhibited more upregulated genes, including *PIN1*, *SAURs* (Small Auxin-Up RNAs), and *DAO* (Figure 4A). Auxin efflux proteins like *PIN1* are well-documented for their role in establishing polar auxin transport [18,51]. Results from the “DUGAN” fir suggested that enhanced auxin biosynthesis and *PINs* activity in the apical buds of this variant maintain lateral buds in a dormant state [48], which aligns with our findings. *SAURs* respond rapidly to auxin signals, relieving H+-ATPase inhibition by suppressing PP2C-D-type protein phosphatase activity [52,53], while the *DAO* gene is responsible for auxin degradation [21,54].

Based on these observations, we speculate that clone C1 maintains apical dominance through higher IAA levels and more efficient polar transport, leading to longer internodal growth. However, it also has a more precise mechanism for releasing this dominance, allowing lateral bud emergence and avoiding the “DUGAN” phenotype, which lacks lateral branches.

### 4.3. Secondary Hormone Signals Involved in Internodal Growth, Especially Brassinosteroids

Endogenous hormones in plants exhibit a complex mechanism of coordinated changes, and similar phenomena were also observed in our research [55,56]. CTK synthesis is typically suppressed by auxin [57,58]; however, in clone C1, CTK levels in the apical and lateral buds did not appear to be affected by this suppression (Figure 2C,D), despite the significant downregulation of related genes (Figure 5B). We speculate that this may be linked to C1’s need to maintain higher radial growth activity. In contrast, CTK levels in the UP tissues showed patterns opposite to those of IAA (Figure 2C,D), suggesting that auxin may indeed inhibit CKTs in these tissues. Notably, at the S4 stage, a sharp decrease in IAA content in the UP tissues was accompanied by a significant increase in CTK levels (Figure 2C,D). This shift may be related to the release of apical dominance and the initiation of lateral bud growth [21].

Similarly, SL levels in the UP tissues showed an inverse pattern compared to IAA. Chen et al. suggested that the interaction between SLs and auxin can be both positive and negative [47]. Additionally, SLs may also be synthesized in the stem [57]. This process could involve the *DWARF* gene family, key players in the SL biosynthesis pathway [59,60,61]. In our study, we found that *D4* and *D27* homologs were downregulated in the apical bud tissues (Figure 5C,D). Two *PDR1* homologs, which were expressed only in the UP tissues, were also observed downregulating (Figure 5C). *PDR1* is one of the few known SL transporters [62] and is typically co-expressed with *DAD1* [63]. Studies on the *PaPDR1* mutant have shown that its polar localization in root epidermal cells mediates the upward transport of SLs [64]. Therefore, it is reasonable to speculate that SLs involved in lateral bud growth in *C. lanceolata* primarily depends on root-to-shoot transport, rather than local stem synthesis.

BRs appear to play a significant role as secondary hormone signals in internodal growth, as indicated by differences in BR content (Figure 2F) and the number of DEGs (Figure 5D). And the “brassinosteroid biosynthesis” was the only pathway directly related to endogenous hormone synthesis significantly enriched across all three tissue types (Figure 4C–E). We observed upregulation of *UGT85A1*, which is believed to regulate BR activity by glucosylating, increasing their stability but rendering them inactive [65]. Additionally, *UGT85A1* is also known to regulate the balance of *trans*-zeatin, a type of CTK [66]. In the context of lateral bud outgrowth in *C. lanceolata*, we believe its expression aligns more with changes in BR levels. Another key upregulated gene, *BAS1*, converts active BRs into inactive forms through 26-hydroxylation [67]. These indicate that in clone C1, multiple BR degradation pathways are active, collectively limiting BR levels.

### 4.4. Very Long-Chain Fatty Acids Influence Internodal Growth by Regulating Hormone Signaling

The very long-chain fatty acids (VLCFAs) are integral components of complex lipids, such as sphingolipids and phospholipids, found in biological membranes [68]. These fatty acids are crucial for maintaining membrane fluidity, thickness, and function, while also serving as precursors for cutin, suberin and waxes.

Roudier et al. found that defective VLCFAs elongation in the eld1 mutant disrupted the auxin gradient in meristematic tissues [69], which is essential for normal growth and cell differentiation. The application of exogenous VLCFAs restored both lateral root formation and auxin polarity distribution in the mutant, suggesting that VLCFAs play a role in the localization and function of auxin transport proteins like PIN1. This regulation likely occurs through alterations in membrane composition and fluidity, influencing the transmission of auxin signals [70]. In our study, we observed upregulation of *PIN1* homologous genes in the UP tissues (Figure 5A), which may indicate differences in the membrane structure and function of conductive tissues. These VLCFA-mediated changes could affect the polar transport of auxin.

WGCNA revealed that the gene sets correlated with endogenous hormone dynamics in the pink module were frequently enriched in pathways related to fatty acid metabolism (Figure 6C). The core hub gene in this module, *ECR* (Figure 7A), encodes a key enzyme in the fatty acid elongation complex, catalyzing the reduction of enoyl-CoA, thus extending fatty acid chains [70]. The VLCFA functional module, with *ECR* as its core, exhibited generally lower expression level in clone C1 than in C11, including biosynthetic genes such as *KCS1* (Figure 7B, Table S6). Although most genes did not reach statistical significance (FDR < 0.05), their highly consistent downregulation pattern suggests that VLCFA content or chain length may be lower in C1.

Very long-chain fatty acids (VLCFAs) are generally recognized as important components of membrane fluidity, and appropriate levels of VLCFAs facilitate the function of membrane proteins such as the auxin efflux carrier PIN [69]. However, from either a quantitative or structural perspective, aberrant VLCFA levels may lead to excessive aggregation of membrane lipid rafts, increased membrane rigidity, or excessive deposition of cuticular/wax barriers, thereby inhibiting IAA transport and cell elongation [71]. Therefore, the relationship between VLCFAs and internode elongation may follow an inverted U-shaped curve: moderate VLCFA module activity in C1 likely falls within the optimal range for internode growth, whereas excessively high VLCFA module activity in C11 falls into the inhibitory range. Notably, the BR-responsive transcription factor gene *BEH4* (a member of the *BES1/BZR1* family) was significantly downregulated in C1 (Figure 5D) and is a putative transcription factor of *KCS1* [72]. Together with the observation that the BR biosynthesis pathway was significantly downregulated in all three tissues (Figure 4C–E), we speculate the existence of a BR-*BES1/BZR1*(*BEH4*)-*KCS1* regulatory axis that modulates polar auxin transport via VLCFAs, which is verified in cotton [72] but not reported in *C. lanceolata* yet.

Although most VLCFA-related genes, including the putative hub gene *ECR*, did not show significantly differential expression in the pink module, it remains an open question whether they play a role in mediating membrane function and structure, thereby coordinating the flow of various endogenous hormone signals. The significant enrichment of pathways associated with membrane function and fatty acid metabolism also indicates a substantial presence of DEGs. Notable examples include cutin, suberin, and wax biosynthesis, as well as arachidonic acid, linoleic acid, and alpha-linolenic acid metabolism, sulfur metabolism, and terpenoid backbone biosynthesis (Figure 4C–E). However, these DEGs were not grouped within the pink module, which is strongly associated with the detected endogenous hormones. This indicates that variations in VLCFA content or composition are present between the two clones. We propose that these differences may affect membrane fluidity [73], thereby influencing the transmission of endogenous hormone signals. Such changes could alter the distribution patterns of IAA and other hormones across various tissues, ultimately impacting the timing of lateral bud initiation and the growth vigor of the apical bud. Together, these factors could shape the differences in internode growth efficiency observed between the clones.

### 4.5. A Potential Endogenous Hormone Distribution and Regulatory Pattern in C. lanceolata Suitable for Knot-Free Timber Cultivation

**Figure 8:**
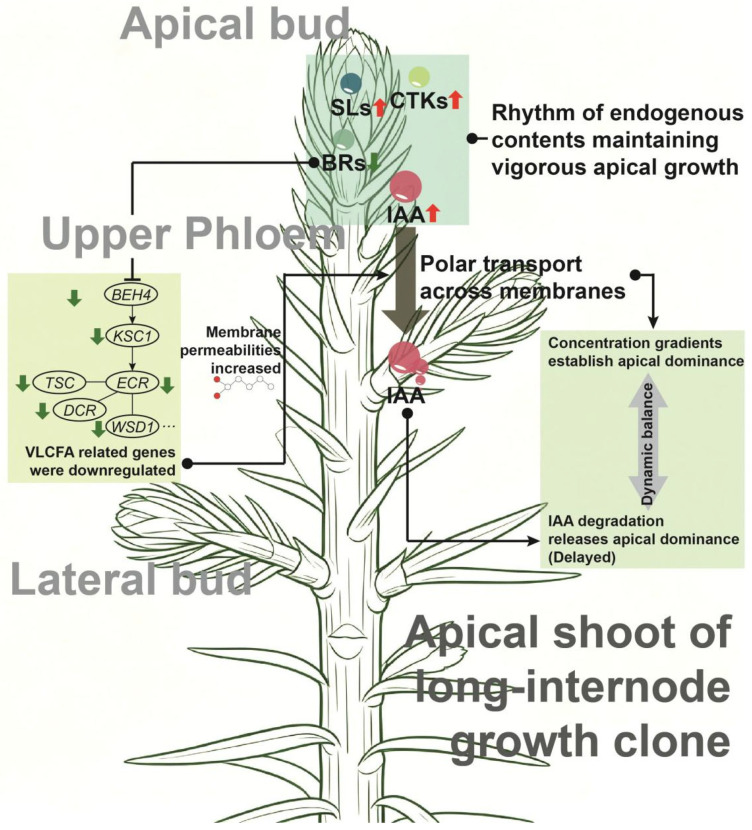
A potential model for the distribution and regulation of endogenous hormones in *C. lanceolata* with delayed lateral branch initiation and longer internodes. A → B denotes positive regulation of B by a change in A, and A ⊣ B denotes negative regulation of B by a change in A.

Based on our findings, we propose a model of endogenous hormone distribution and regulation pattern in *C. lanceolata* clones suited for knot-free timber cultivation (Figure 8). These resources likely exhibit strong auxin biosynthesis and transport ability, establishing a concentration gradient that maintains apical dominance for an extended period relatively. Concurrently, sufficient auxin levels in the apical buds, coupled with relatively high CTK levels, support longer internodal growth. Lateral bud formation is delayed by the combined influence of apical dominance and SLs transported from the roots. After this delay, efficient IAA degradation in the lateral meristems releases apical dominance, allowing lateral bud initiation and development. The regulation of hormone transport may involve VLCFAs, further influencing hormone distribution and signaling during lateral bud growth. This potential model underscores the critical balance between hormone synthesis, transport, and degradation as key to optimizing the development of knot-free timber.

The limitations of this study lie in the limited precision of hormone level measurement, which only conducts a preliminary analysis. We will consider adopting liquid chromatography-mass spectrometry (LC-MS) to conduct more precise quantitative analysis of hormone concentrations. At the same time, for the role of VLCFAs in the regulatory network that controls the internode growth of Chinese fir, more specific evidence is needed. A comprehensive multi-omics approach combining lipidomics, transcriptomics and proteomics may be considered for future research.

## 5. Conclusions

This study examined the hormonal regulation mechanisms involved in lateral bud initiation in *C. lanceolata*. By comparing growth and branching traits, we selected two clones with significant differences in branching characteristics. The endogenous levels of IAA, cytokinins, strigolactones, and brassinosteroids varied notably between the two clones across apical buds, upper phloem, and lateral buds. RNA-seq analysis revealed distinct differences in gene expression related to the biosynthesis, degradation, and transport of these hormones. Additionally, very long-chain fatty acids in the upper phloem may influence hormone transport and distribution. These findings provide a preliminary understanding of hormonal regulation in lateral bud development, offering valuable insights for breeding knot-free timber varieties of *C. lanceolata*.

## Data Availability

RNA-Seq data were deposited into the Gene Expression Omnibus database under accession number GSE297964 and are available at the following URL: https://www.ncbi.nlm.nih.gov/geo/query/acc.cgi?acc=GSE297964 (accessed on 23 May 2025). Data supporting the findings of this study are available within the paper and its Supplementary Information.

## Supplementary Materials

The following supporting information can be downloaded at: https://www.mdpi.com/article/10.3390/plants15091411/s1, Table S1: List of 13 Clones; Table S2: All DEGs FPKM value; Table S3: Information of KEGG enrichment; Table S4: ID list of the genes in pink module; Table S5: KEGG enrich analysis of pink module genes; Table S6: Top 20 (Degree) genes in pink module; Figure S1: Persistent nature of lateral branches resist natural shedding in *C. lanceolata*; Figure S2: Geographic position of the sampling plots; Figure S3: Comparative analysis of growth and branching characteristic of 13 sampled *C. lanceolata* Clone.

## Abbreviations

IAA	Indole-3-acetic acid
CTK	Cytokinin
SL	Strigolactone
BR	Brassinosteroids
AB	Apical bud
LB	Lateral bud
UP	Upper Phloem
DEG	Differential expressed genes
WGCNA	Weighted gene co-expression network analysis
KEGG	Kyoto Encyclopedia of Genes and Genomes
FPKM	Fragments Per Kilobase of exon model per Million mapped fragments
VLCFA	Very long-chain fatty acid
