# Supplementary figures and images for: Enhanced Polar Auxin Transport and Reduced Brassinosteroid Activity Drive Internode Elongation in Chinese Fir (Cunninghamia lanceolata)
Source: Plants (Basel). 2026 May 5;15(9):1411. doi: 10.3390/plants15091411 (PMC13164657; doi:10.3390/plants15091411)

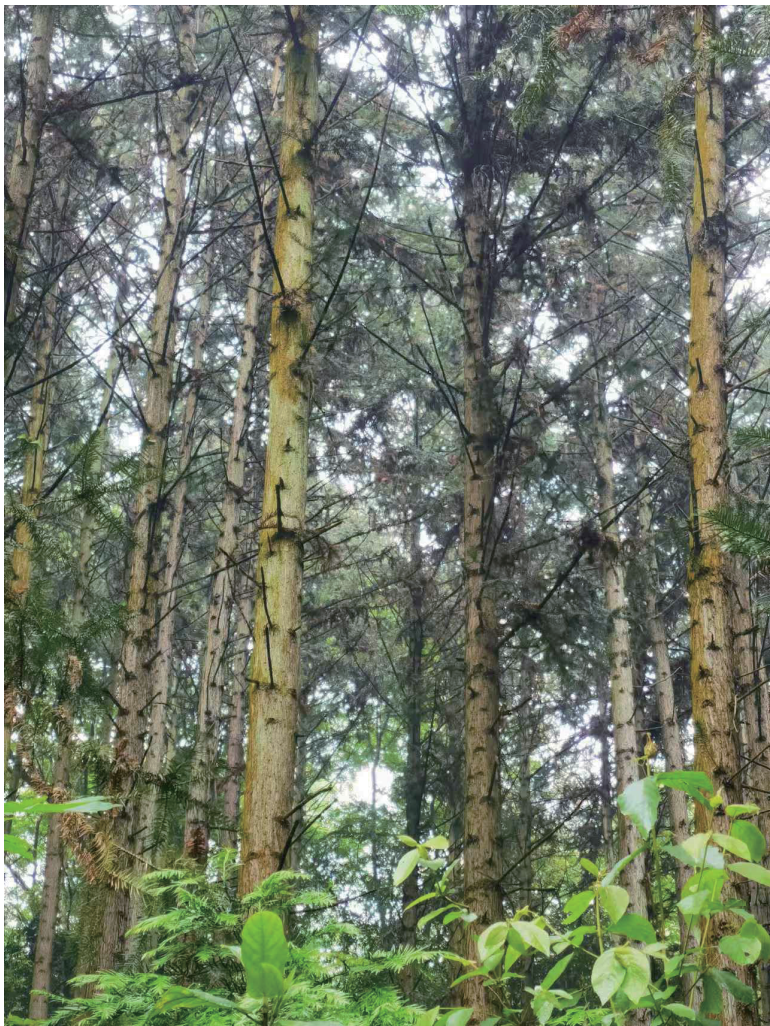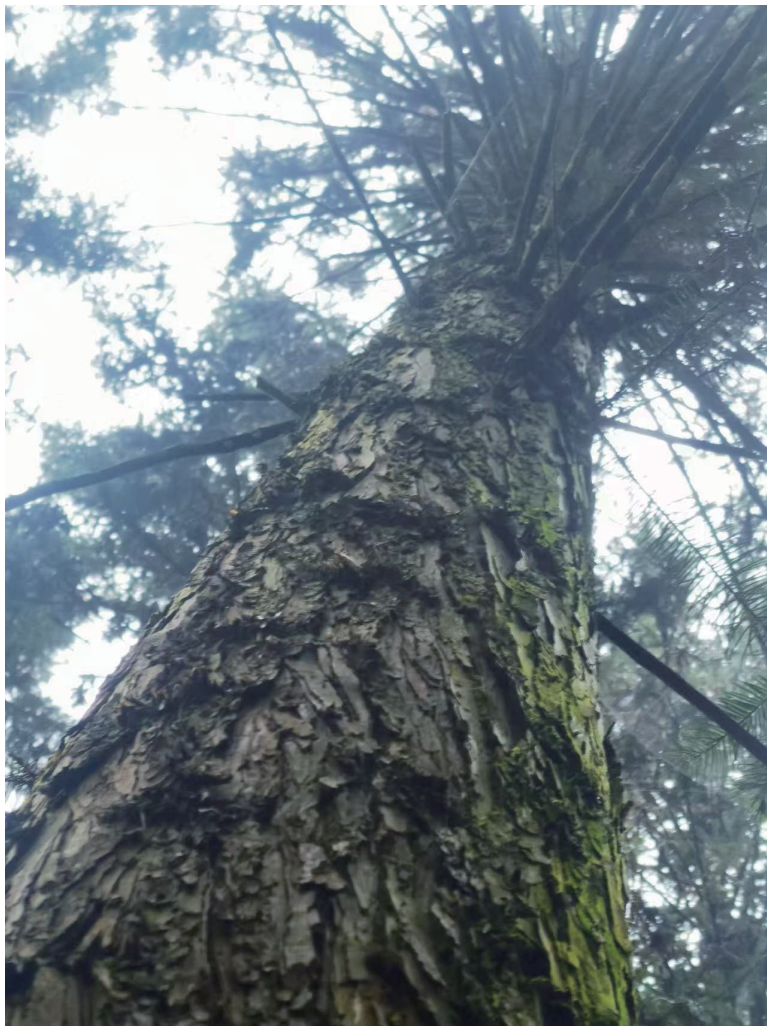

Supplement: Supplementary file 1 [file plants-15-01411-s001.zip › Figure_S1.pdf]

Map of Fujian Province with a Marked Point

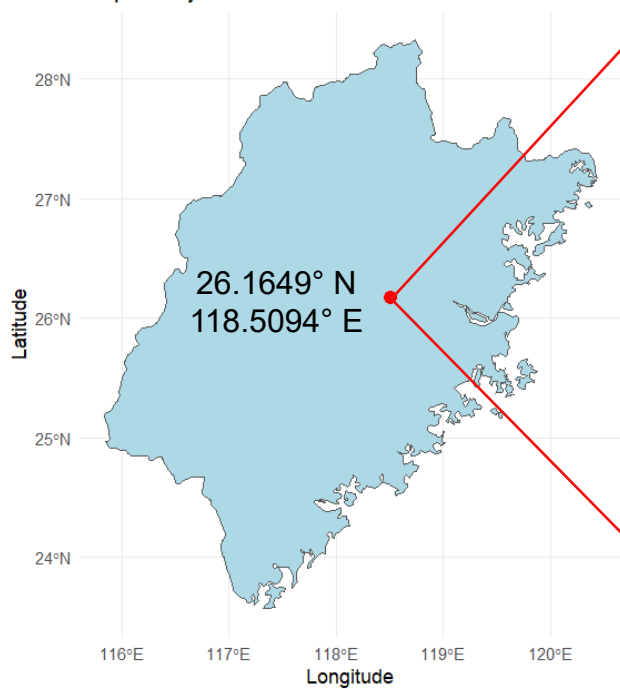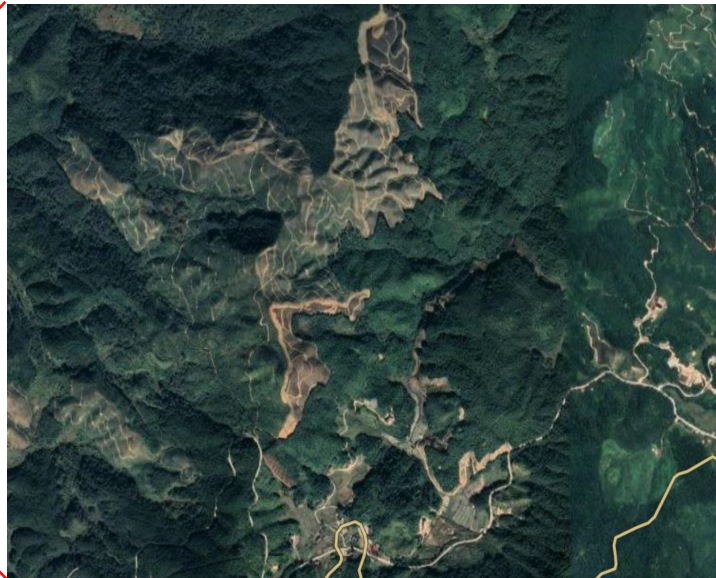

Supplement: Supplementary file 1 [file plants-15-01411-s001.zip › Figure_S2.pdf]

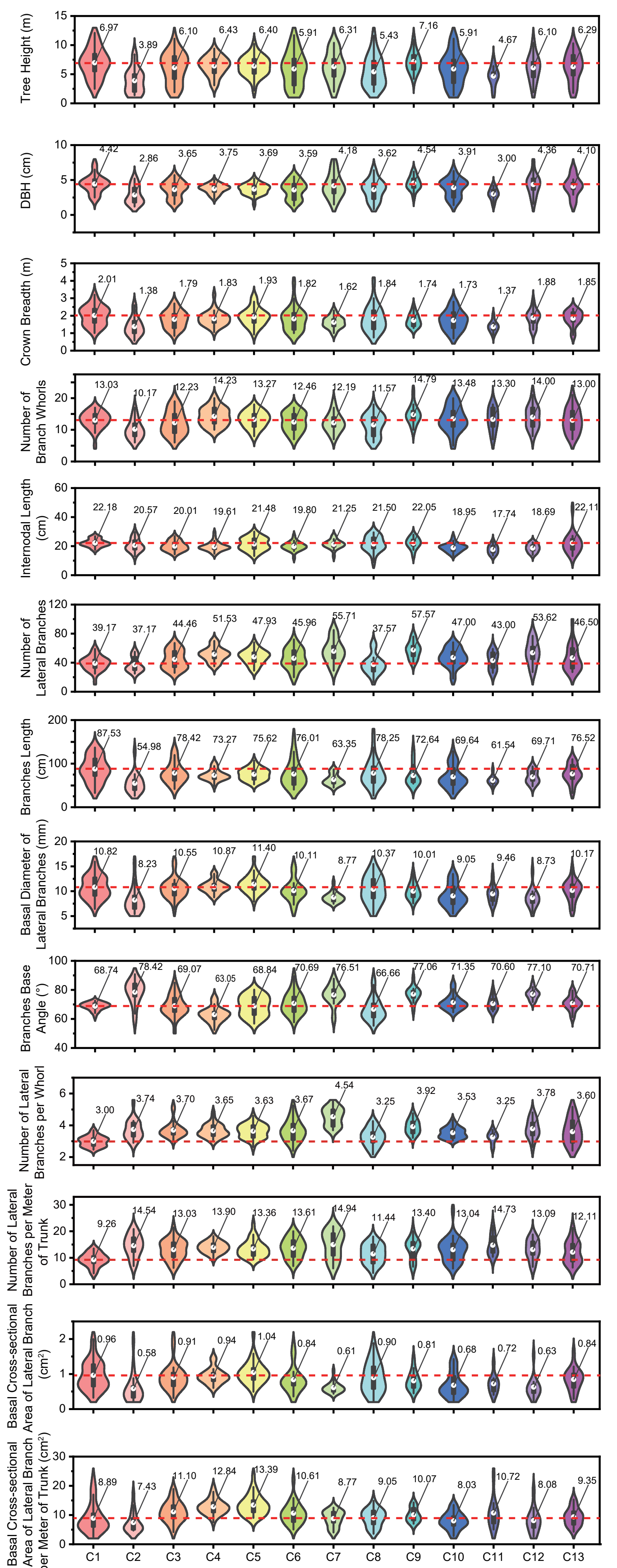

Supplement: Supplementary file 1 [file plants-15-01411-s001.zip › Figure_S3.pdf]
